# Sepsis-Associated Acute Kidney Injury in Critically Ill Children: Incidence and Outcomes

**DOI:** 10.3390/jcm13226720

**Published:** 2024-11-08

**Authors:** Mohammad A. Shalaby, Khalid A. Alhasan, Ibrahim A. Sandokji, Amr S. Albanna, Zahrah Almukhtar, Hind Khalifa Elhaj, Khaled Alwadai, Abdulaziz Bahassan, Mohamad-Hani Temsah, Rupesh Raina, Jameela A. Kari

**Affiliations:** 1 Pediatric Department, Faculty of Medicine, King Abdulaziz University, Jeddah 21589, Saudi Arabia; mshalaby1977@hotmail.com (M.A.S.); elhaj.hind@gmail.com (H.K.E.); alwad3i.711@gmail.com (K.A.); ambahassan@kau.edu.sa (A.B.); 2 Pediatric Nephrology Unit, Pediatric Nephrology Center of Excellence, King Abdulaziz University Hospital, King Abdulaziz University, Jeddah 21589, Saudi Arabia; 3 Pediatric Department, College of Medicine, King Saud University, Riyadh 11451, Saudi Arabia; zahrahmuk@live.com (Z.A.); mtemsah@ksu.edu.sa (M.-H.T.); 4 Kidney & Pancreas Health Center, Organ Transplant Center of Excellence, King Faisal Specialist Hospital & Research Center, Riyadh 12713, Saudi Arabia; 5 Pediatric Department, College of Medicine, Taibah University, Madinah 42353, Saudi Arabia; isandokji@taibahu.edu.sa; 6 King Abdullah International Medical Research Center, King Saud Bin Abdulaziz University for Health Sciences, Jeddah 14611, Saudi Arabia; amralbanna@gmail.com; 7 Department of Nephrology, Cleveland Clinic Akron General and Akron Children Hospital, Akron, OH 44307, USA; rraina@akronchildrens.org

**Keywords:** acute kidney injury, pediatric, KDIGO, sepsis

## Abstract

**Background:** Acute kidney injury (AKI) is a major concern in pediatric critical care, often occurring in conjunction with sepsis. This study aimed to identify the incidence, outcomes, and risk factors for AKI in the context of pediatric sepsis. **Methods:** This was a bicentric retrospective cohort study conducted at two university hospitals in Saudi Arabia. All patients aged 1 month to 14 years admitted to pediatric intensive care units (PICUs) with evidence of sepsis between January 2021 and December 2022 were included. AKI was defined and staged according to the Kidney Disease: Improving Global Outcomes (KDIGO) criteria. Demographic, clinical, and laboratory data were collected from electronic medical records. **Results:** 309 patients were included, 38 (12.3%) developed stage 1 AKI, 64 (20.7%) developed stage 2 AKI, and 183 (59.2%) developed stage 3 AKI. Patients with sepsis-associated AKI had significantly longer PICU stays and higher mortality rates than those without AKI (*p* < 0.01). Inflammatory markers and certain medications were associated with increased AKI risk. Factors independently associated with stage 3 AKI include younger age, positive blood culture, gentamycin use, and higher SOFA score. **Conclusions:** Sepsis-associated AKI is a common and serious complication in critically ill children, contributing to increased morbidity and mortality. Identification of specific risk factors may facilitate early recognition and targeted interventions to mitigate the impact of AKI in this vulnerable population.

## 1. Introduction

Acute kidney injury (AKI) remains a significant concern in pediatric critical care [1]. Formerly recognized as acute kidney failure, AKI is a rapid decline in kidney function, characterized by an increase in serum creatinine levels, a decrease in urine output, or both, ranging from a modest reduction in kidney function to complete renal failure [2]. This impairment in kidney function disrupts the delicate balance of fluids, electrolytes, and acid–base homeostasis, which are essential for the management and outcomes of critically ill children [3]. In 2012, the kidney disease improving global outcomes (KDIGO) workgroup introduced a detailed AKI classification system [1]. This system has been extensively validated for its sensitivity and prognostic accuracy in identifying various stages of AKI and predicting patient outcomes [2]. The risk of mortality in AKI patients increases with the progression of the condition, with stages 2 and 3 associated with significantly higher risks of adverse outcomes [3]. Persistent AKI, which does not resolve promptly, poses an even greater risk of poor outcomes, including chronic kidney disease and increased mortality, compared to transient AKI [4].

AKI is particularly common among critically ill children, often occurring in conjunction with sepsis and requiring intensive care support. In pediatric intensive care units (PICUs), the leading causes of AKI include sepsis, multiple organ dysfunction syndrome, complicated cardiac surgeries, and exposure to nephrotoxic medications, all of which complicate the management and outcomes of these young patients [5].

The link between sepsis and AKI is especially evident in critically ill children [6,7]. In neonates and children, sepsis often underlies AKI, contributing to more severe stages of kidney injury and increasing morbidity and mortality rates [8,9,10]. Sepsis is a common trigger of AKI, especially in critically ill pediatric patients, further complicating treatment and outcomes.

Sepsis, a life-threatening condition caused by the body’s extreme response to infection, triggers a cascade of changes that can lead to multi-organ failure.

The systemic inflammatory response syndrome (SIRS) criteria have been widely used to define sepsis, especially if combined with a calculation of the sequential organ failure assessment (SOFA) score to identify organ dysfunction. There are other definitions which emphasize organ dysfunctions rather than inflammation alone, like the Sepsis-3 criteria mainly for adult patients, and the Phoenix sepsis score for pediatric patients [11].

The mechanisms behind sepsis-induced AKI are still not fully understood. Current understanding suggests that septic AKI arises from an abnormal immune response and widespread inflammation, hemodynamic changes, dysfunction of renal microvascular endothelial cells, and damage to renal tubular epithelial cells [12].

Early management of sepsis, focusing on restoring organ perfusion through fluid resuscitation and vasoactive medications, is crucial in mitigating these risks [13]. Pediatric patients with AKI, especially when associated with sepsis, often face longer durations of mechanical ventilation, extended hospital stays, progression to chronic kidney disease (CKD), and increased mortality rates [14].

Over the years, several prediction models for AKI in sepsis patients have been developed for the adult population [15]. These models help in identifying patients at high risk and guiding early interventions [16]. However, there are fewer prediction models tailored specifically for pediatric patients that needs more validation [17].

This research aims to identify measurable risk factors for AKI in the context of pediatric sepsis. Identifying these risk factors is crucial for timely and effective intervention, which could potentially improve outcomes by reducing the severity or preventing the onset of AKI.

## 2. Subject and Methods

### 2.1. Data Collection

Our study involves two sites: a retrospective cohort study from the two largest university hospitals in KSA (King Abdulaziz and King Saud universities) in Jeddah and Riyadh, which cover two of the largest geographical regions in the country. Medical records of all patients admitted to pediatric intensive care units (PICUs) at both hospitals were reviewed.

### 2.2. Inclusion and Exclusion Criteria

We reviewed all patients admitted to pediatric intensive units. All patients aged 1 month to 14 years and with evidence of sepsis in the period between the 1st of January 2021 till the 31st of December 2022 were included in our study. All patients less than 1 month of age with evidence of CKD, or those with insufficient clinical data were excluded.

All required information was extracted from the electronic records system in a fully informative designated Excel sheet. The target information included the following: demographic data (age, gender, weight, height, date of admission, and date of discharge), clinical and laboratory data related to sepsis (admitting diagnosis, vital signs, presenting symptoms, blood gases, SOFA score, use of nephrotoxic medications, and use of inotropes), and clinical and laboratory renal data (baseline creatinine, baseline glomerular filtration rate, highest creatinine during initial seven days of PICU admission, UOP recorded during PICU admission, and use and modalities of renal replacement therapy).

### 2.3. Definitions

Acute kidney injury was defined according to the KDIGO creatinine criteria [1]. The KDIGO consensus defines AKI as an increase in serum creatinine level to ≥0.3 mg/dL (≥26.5 mmol/L) within 48 h, increase in serum creatinine by ≥1.5 times from baseline, or decrease in urine volume to ≤0.5 mL/kg/h. It classifies AKI into three stages based on the magnitude of changes in serum creatinine and/or UOP levels, as follows: stage 1, increase of serum creatinine to ≥0.3 mg/dL (≥26.5 mmol/L) or by 1.5 to 1.9 times from baseline, and/or decrease in UOP to less than 0.5 mL/kg/h for 6–12 h; stage 2, increase of serum creatinine by 2 to 2.9 times from baseline and/or decrease in UOP to less than 0.5 mL/kg/h for >12 h; and stage 3, increase of serum creatinine to ≥353.6 mmol/L or by greater than three times from baseline, decrease in GFR to less than 35 mL/min/1.73 m^2^, decrease in UOP to less than 0.5 mL/kg/h for >24 h, or anuria for >12 h [1]. The estimated glomerular filtration rate (eGFR) was calculated using the modified Schwartz formula [18]. Due to the lack of hourly urine output (UOP) data, accurate measurements were not available for all patients in our retrospective cohort study. This issue is consistent with many published AKI studies that rely on serum creatinine-based definitions. Using creatinine-based criteria may have the advantage of preventing the overestimation of AKI incidence, which can arise from technical difficulties and errors in urine collection and UOP recording [19].

Baseline creatinine was defined as the last creatinine within the previous 6 months prior to PICU admission; for those patients admitted for the first time with no previous creatinine, we used the average GFR according to the age, gender, and height of the child [20].

Sepsis was defined using the SIRS criteria: body temperature above 38 °C or below 36 °C, heart rate greater than 90 beats per minute, respiratory rate greater than 20 beats per minute or carbon dioxide partial pressure below 4.3 kPa, neutrophilia above 12,000/mm^3^ or neutropenia below 4000/mm^3^ with 10% or more of non-segmented peripheral blood neutrophils, and/or clinical symptoms with positive culture (blood, urine, and/or CSF) [21].

To assess associated organ dysfunction we used the SOFA score: The SOFA score, previously known as the sepsis-related organ failure assessment score, is used to track a person’s status during a stay in an intensive care unit (ICU) to determine the extent of a person’s organ function or rate of failure. The score is based on six different scores, one each for the respiratory, cardiovascular, hepatic, coagulation, renal, and neurological systems, with a higher SOFA score indicating greater organ dysfunction [22].

### 2.4. Outcome Measures

We referred to the length of PICU admission/day, length of hospital stays, incidence of mortality during PICU admission, and discharge with abnormal kidney function as an indicator of outcome.

Ethical Considerations: This study was approved by the Institutional Review Boards (IRBs) of both participating hospitals, with reference numbers 290-22 and 24/1426/IRB. All procedures followed the ethical standards of the responsible committees on human research and conformed to the principles outlined in the Declaration of Helsinki. All patient data were handled in strict accordance with institutional and national data protection guidelines. All data were anonymized, and no identifiable information was included in the study.

Statistical analysis: All analyses were performed using STATA (StataCorp. 2011, Stata Statistical Software, Release 12, College Station, TX, USA: StataCorp LP) software. The proportion and mean for dichotomous and continuous variables, respectively, were measured to describe patients’ characteristics. The risk of AKI and related outcomes including in-hospital mortality, renal impairment at discharge, and length of stay in the hospital and ICU, were compared between patients with different stages of acute kidney injury using chi-square or Fisher’s exact tests for categorical variables, and one-way analysis of variance (ANOVA) and Kruskal–Willis tests for continuous variables. Logistic regression analysis was performed to determine the independent risk factors for severe AKI (stage 3). Multivariate logistic regression analysis was performed including variables that were significantly associated with severe AKI in the univariant regression analysis (age, leukocytosis, thrombocytopenia, anemia, oliguria, positive blood culture, SOFA score, and the use of diuretics, inotropes, amikacin, gentamycin, and vancomycin). Statistical significance was determined using the 95% confidence interval and *p*-value of 0.05.

## 3. Results

A total of 309 patients admitted with severe sepsis were included in the study, of which 24 (7.8%) did not develop AKI, 38 (12.3%) developed stage 1 AKI, 64 (20.7%) developed stage 2 AKI, and 183 (59.2%) developed stage 3 AKI. Table 1 shows the baseline demographic and clinical characteristics of the included patients.

**Figure 1:**
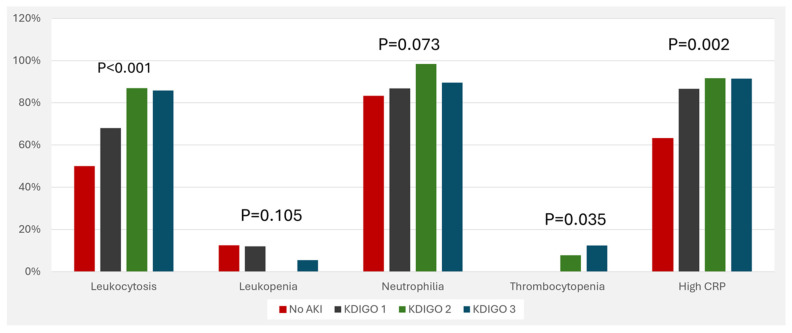
Risk of acute kidney injury with different inflammatory markers. Abbreviations: AKI, acute kidney injury; KDIGO, kidney disease improving global outcomes; CRP, C-reactive protein.

**Figure 2:**
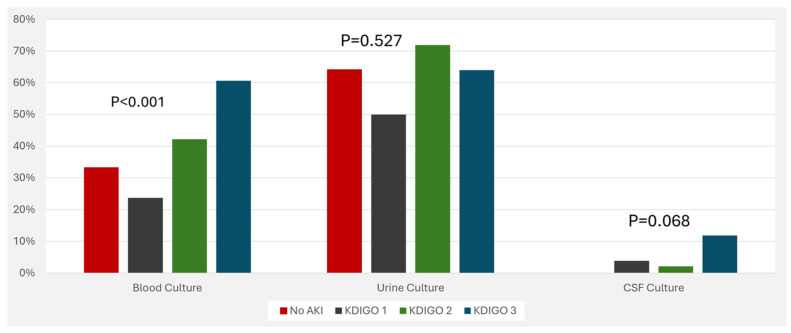
Risk of acute kidney injury among patients with positive culture. Abbreviations: AKI, acute kidney injury; KDIGO, kidney disease improving global outcomes; CSF, cerebrospinal fluid.

**Figure 3:**
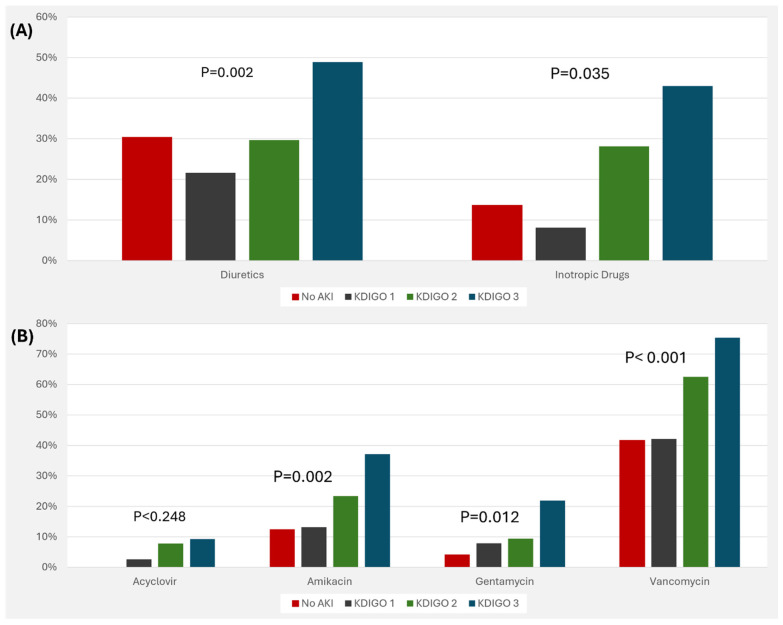
Risk of acute kidney injury with the use of different supportive (**A**) and nephrotoxic (**B**) therapies. Abbreviations: AKI, acute kidney injury; KDIGO, kidney disease improving global outcomes.

The association between inflammatory markers and AKI stages is visualized in Figure 1. In addition, positive blood culture was associated with increased risk of AKI (Figure 2). Medications that were associated with increased risk of AKI include diuretics, inotropic drugs, amikacin, gentamycin, and vancomycin ( Figure 3).

Using multiple regression analysis, the factors that were independently associated with the development of stage 3 AKI included age (OR, 0.990; 95% CI, 0.981–0.997; *p* = 0.011), positive blood culture (OR, 2.2; 95% CI, 1.1–4.46; *p* = 0.026), the use of gentamycin (OR, 2.89; 95% CI, 1.04–8.05, *p* = 0.042), and SOFA score (OR, 1.2; 95% CI, 1.1–1.4; *p* = 0.001), as shown in Table 2.

**Figure 4:**
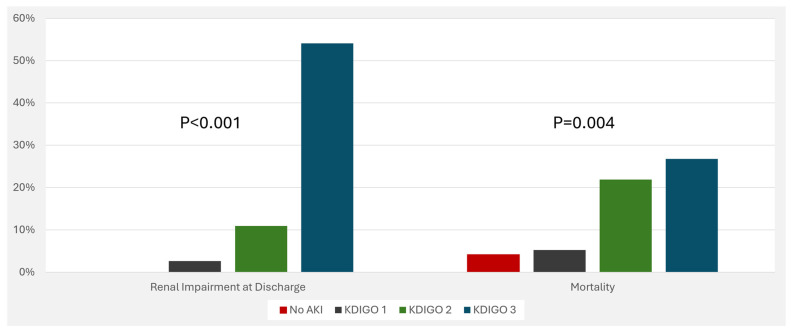
Outcomes (renal impairment at discharge and in-hospital mortality) associated with acute kidney injury. Abbreviations: AKI, acute kidney injury; KDIGO, kidney disease improving global outcomes.

**Figure 5:**
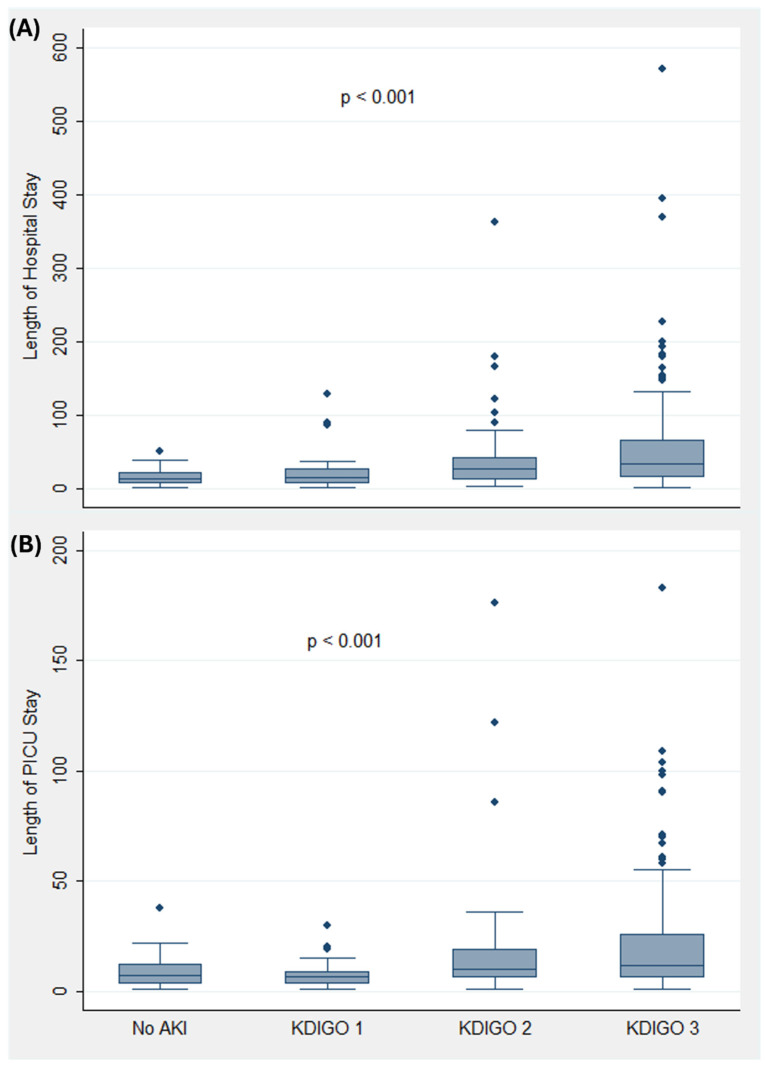
Length of hospital (**A**) and ICU (**B**) stay among patients with acute kidney injury. Abbreviations: ICU, intensive care unit; AKI, acute kidney injury; KDIGO, kidney disease improving global outcomes.

The overall in-hospital mortality among included patients was 21.4% (95% CI: 16.8–26.0). The risk of in-hospital mortality increased as the severity of AKI increased (Figure 4). Other outcomes that were associated with AKI severity were the length of hospital and ICU stay and renal impairment at the time of discharge from the hospital (Figure 4 and Figure 5).

## 4. Discussion

This retrospective, bicentric study reports on the epidemiology and outcomes of sepsis-associated AKI in hospitalized children. Higher SOFA scores, younger age, positive blood cultures, and gentamicin use were independently associated with stage 3 AKI.

The biological mechanisms linking sepsis to AKI involve complex interactions among inflammatory, hemodynamic, and cellular processes. Sepsis triggers a systemic inflammatory response, releasing pro-inflammatory cytokines like IL-6 and TNF-α, leading to endothelial dysfunction and increased vascular permeability. This causes reduced renal perfusion from hypotension and microcirculatory dysfunction, resulting in kidney ischemia. Additionally, nephrotoxic metabolites and medications can further impair renal function. Direct injury to renal tubular cells, exacerbated by apoptosis and mitochondrial dysfunction, contributes to kidney damage. Coagulation abnormalities, including microthrombi formation, obstruct blood flow and promote ischemia. These interconnected mechanisms underscore the critical relationship between sepsis and AKI, emphasizing the need for targeted therapeutic approaches.

The relationship between sepsis and AKI was discussed in a well-titled review article, “Sepsis and AKI: A Two-Way Street” [23]. In addition to the fact that AKI risk increases in patients with sepsis, recent evidence suggests that the development of AKI, in turn, increases the risk of developing sepsis. This was confirmed by a multicenter study including 618 critically ill patients with sepsis, in which 40% developed sepsis a median of 5 days after AKI [24]. In pediatrics, a study investigating AKI in a cohort of 5538 critically ill children who did not have sepsis at the time of ICU admission found that higher stages of AKI were associated with twice the odds of developing sepsis [25].

We have shown that higher SOFA scores were associated with higher stages of AKI and, in a multivariate regression analysis, were independently associated with higher mortality rates. The pediatric SOFA score is a well-known clinical tool to assess severity and predict outcomes in critically ill patients. It was adapted to pediatric reference ranges and developmental stages in children [26]. In a study on critically ill hospitalized children, higher SOFA scores were associated with increased severity and risk of AKI [27]. In a large cohort study of 6303 pediatric patients in the intensive care unit, the pediatric SOFA score showed an excellent ability to predict in-hospital mortality with an area under the curve of 0.94 (95% CI: 0.92–0.95) [28].

A large multicenter study of 2121 infants with AKI found that diuretic use, which can exacerbate AKI, was more common in smaller birth weight and younger gestational-age infants [29].

The SPROUT study, involving 128 PICUs in 26 countries, found that severe AKI was present in approximately 20% of pediatric patients with severe sepsis [9].

Our study shows that higher stages of AKI are associated with worse outcomes, including higher mortality, longer length of stay, the need for kidney support therapies, and acute kidney disease. This is consistent with the previously mentioned important study (SPROUT), which found that more than twice as many patients with severe AKI died or developed new moderate disability compared to those with no or mild AKI. Severe AKI was independently associated with death or new moderate disability.

In Saudi Arabia, only a few studies have investigated the epidemiology of AKI in children [30]. A prospective cohort study of three tertiary hospitals in Saudi Arabia on the epidemiology of AKI in hospitalized children found that AKI affected one-third of children admitted to the ICU and showed a six-fold increase in mortality, even after adjustment for baseline demographics [31]. During the recent COVID-19 pandemic, a multicenter cohort study was conducted in three tertiary hospitals in Saudi Arabia on the epidemiology of AKI in hospitalized children with COVID-19 [32]. The development of AKI was associated with higher mortality rates and residual renal impairment.

In our cohort, laboratory markers of inflammation were associated with higher stages of AKI, such as leukocytosis and elevated CRP levels. C-reactive protein (CRP) contributes to the development of AKI by binding to its receptors, CD32 and CD64, which subsequently activate signaling pathways such as nuclear factor-kappa B (NF-κB) and transforming growth factor-beta (TGF-β)/Smad3 [33]. This activation leads to renal inflammation and macrophage activation, promoting G1 cell cycle arrest and inhibiting autophagy, both of which contribute to kidney damage. Leukocytosis indicates active inflammation that could exacerbate AKI [34,35]. In a study by Formeck et al. on critically ill children with AKI, leukocytosis was identified as a marker of severe systemic response associated with sepsis and the development of AKI [25]. Likewise, CRP, an acute-phase reactant, was found to correlate with the severity of sepsis and organ dysfunction, including AKI and higher mortality in pediatric sepsis [26,36].

Thrombocytopenia develops in critically ill children in consumptive conditions such as disseminated intravascular coagulopathy (DIC) [37]. In a study focused on critically ill children, thrombocytopenia was associated with increased mortality in children with AKI; however, in a multivariate regression analysis, it was not independently associated with worse outcomes [38]. Another study examining the role of platelet dysfunction in children with sepsis found that thrombocytopenia is associated with higher mortality [39]. Moreover, thrombocytopenia was negatively correlated with the pediatric risk of mortality (PRISM III) score, a clinical tool to assess the severity of illness. This agrees with our results, where we noted more thrombocytopenia in higher stages of AKI but not in the multivariate analyses, indicating its role as a marker of systemic inflammation and coagulopathy.

Body volume status and diuresis play critical roles in the outcomes of sepsis-associated AKI. Theoretically, diuretics help support the kidney in keeping the euvolemic state; however, they could, in turn, worsen the kidney injury [40]. In our cohort, we found that the use of diuretics was associated with worse AKI stages. In a study reviewing 1685 pediatric admissions with severe sepsis or septic shock, the use of diuretics was associated with higher rates of major adverse kidney events defined as mortality, use of kidney support therapy, or persistent kidney dysfunction (59% vs. 35%, *p* < 0.05) [41]. Younger infants with growing kidneys are at an even higher risk of kidney injury due to diuretic use in AKI settings. In a large multicenter study from 46 US hospitals, including 2121 infants with AKI, diuretic use was more common in smaller birth weight and younger gestational-age infants [42]. It was independently associated with higher mortality rates; HR: 2.76, 95% CI: 1.98, 3.86 and HR: 2.38, 95% CI: 1.77, 3.22 for short (<5 days) and prolonged (≥5 days) diuretic use, respectively.

## 5. Conclusions

In this bicentric study, we have shown the significance of using the SOFA score in the assessment and monitoring of children with sepsis at risk of AKI. Our data highlight the association between important factors such as younger age, positive blood culture, use of gentamycin, laboratory parameters (high CRP, leukocytosis, thrombocytopenia), and higher SOFA score and the incidence of higher stages of AKI. Children with sepsis-associated AKI have worse outcomes. These findings suggest that a multi-faceted approach considering both clinical and laboratory parameters could inform more targeted and aggressive management strategies to reduce mortality and long-term renal impairment. Future research should focus on developing and validating predictive models that incorporate these risk factors to help in the early detection of AKI and further intervention in this vulnerable age group.

### Limitations of Study

To our knowledge, this is the largest study on pediatric sepsis-associated AKI in children in Saudi Arabia. However, we acknowledge several limitations, including the constraints of being a retrospective study, which affects the availability of clinical and laboratory variables, as well as potential confounding factors not accounted for in the analysis that could further impact our findings. We used (SIRS) criteria to define sepsis and the SOFA score for organ dysfunctional assessment. However, we recommend testing the clinical applicability of other updated definitions of sepsis in pediatric populations.

We relied on serum creatinine to define AKI without including urine output data, as that could have influenced AKI staging and its associations with outcomes. However, it might have an advantage in avoiding an overestimation of this incidence due to the associated technical difficulties and calculation errors of UOP in pediatric patients. Thus, future studies should adopt a prospective design to incorporate clinical, laboratory, novel technologies, and biomarker evaluations [43,44]. Additionally, more extended follow-up studies could help in understanding the long-term outcomes of sepsis-associated AKI in children.

## Figures and Tables

**Table 1:** Baseline characteristics.

Characteristics	No AKI	KDIGO 1	KDIGO 2	KDIGO 3	*p* Value
Male Gender No. (%)	13 (54.2)	13 (34.2)	35 (54.7)	104 (56.8)	0.088 #
Age in month, Mean (SD)	52.9 (50.1)	59.2 (51.9)	51.3 (51.5)	37.3 (44.4)	0.019
Median (IQR)	48 (11–82)	42 (10–108)	22 (8–96)	23 (5–60)	0.020
Fever No. (%)	6 (25.0)	13 (34.2)	16 (25.0)	56 (30.6)	0.713 #
Respiratory distress No. (%)	8 (33.3)	12 (31.6)	19 (29.7)	35 (19.1)	0.118 #
Neuromuscular symptoms No. (%)	5 (20.8)	4 (10.5)	11 (17.2)	23 (12.6)	0.527 #
GI symptoms No. (%)	2 (8.3)	3 (7.9)	2 (3.1)	17 (9.3)	0.424 *
BMI, Mean (SD)	15.3 (5.0)	16.3 (5.0)	14.2 (4.3)	14.8 (4.4)	0.129
Median (IQR)	14.1 (13–15)	15.8 (13–18)	13.8 (11–17)	14.4 (12–16)	0.150
Systolic BP, Mean (SD)	125 (25)	127 (20)	124 (20)	123 (18)	0.600
Median (IQR)	121 (111–145)	126 (113–138)	124 (111–135)	123 (111–132)	0.573
Diastolic BP, Mean (SD)	82 (19)	83 (17)	80 (17)	79 (17)	0.592
Median (IQR)	78 (72–93)	84 (72–94)	86 (66–94)	79 (66–91)	0.554
Hypoxia No. (%)	2 (8.3)	3 (7.9)	7 (10.9)	8 (4.4)	0.205 *
Hypervolemia No. (%)	1 (6.3)	2 (8.0)	6 (13.0)	26 (20.5)	0.288 *
Oliguria No. (%)	0	0	4 (8.7)	19 (15.1)	0.057 *
24 h UOP (mL/kg/h), Mean (SD)	2.1 (1.0)	2.0 (1.2)	1.8 (1.1)	2.1 (1.2)	0.557
Median (IQR)	2.3 (1.1–2.8)	1.7 (1.1–2.8)	1.8 (1.1–2.4)	1.2–2.7)	0.449
Anemia No. (%)	17 (70.8)	24 (63.2)	53 (82.8)	160 (87.4)	0.002 #
TLC Mean (SD)	16.2 (12.1)	15.6 (7.7)	21.1 (9.2)	20.4 (12.5)	0.114
Median (IQR)	14 (9.3–19)	16 (9.5–22)	18 (15–26)	18 (14–25)	0.049
Neutrophil, Mean (SD)	11.5 (9.9)	11.3 (6.8)	14.7 (7.1)	12.9 (8.1)	0.149
Median (IQR)	8 (6–14)	11 (6–15)	14 (10–18)	12 (7–18)	0.046
Platelet, Mean (SD)	392 (226)	430 (170)	372 (176)	384 (244)	0.621
Median (IQR)	365 (260–432)	408 (326–501)	391 (265–462)	362 (213–515)	0.343
ESR Mean (SD)	29.5 (27.8)	47.2 (38.9)	32.3 (26.5)	37.8 (31.0)	0.734
Median (IQR)	30 (4–54)	36 (18–87)	23 (13–49)	32 (7–61)	0.866
CRP Mean (SD)	57.3 (78.8)	110 (105)	77.7 (69.7)	111 (91.7)	0.011
Median (IQR)	19 (3–88)	84 (17–152)	51 (21–130)	91 (37–162)	0.005
Baseline creatinine, Mean (SD)	26.0 (10.5)	26.3 (27.3)	26.2 (48.2)	13.9 (23.1)	0.007
Median (IQR)	21 (19–33)	21 (15–27)	14 (12–21)	8 (5–14)	<0.001
Highest creatinine, Mean (SD)	32.5 (13.5)	45.2 (47.8)	61.9 (111)	95.3 (160)	0.032
Median (IQR)	28 (24–42)	34 (26–48)	36 (27–50)	47 (31–82)	<0.001
RRT use No. (%)	0	0	18 (28.1)	65 (35.5)	<0.001
Type of RRT No. (%)					0.737 *
Hemodialysis	0	0	18 (100)	59 (90.8)	
CRRT	0	0	0	4 (6.2)	
Peritoneal dialysis	0	0	0	1 (1.5)	
More than one modality	0	0	0	1 (1.5)	
SOFA Score, Mean (SD)	4.1 (2.9)	5.2 (3.2)	4.8 (3.0)	7.1 (3.6)	<0.001
Median (IQR)	3.5 (2–6)	5 (3–7)	5 (3–6)	7 (5–9)	<0.001

Abbreviations: AKI, acute kidney injury; KDIGO, kidney disease improving global outcomes; GI, gastrointestinal; BMI, body mass index; BP, blood pressure; UOP, urine output; TLC total leukocyte count; ESR, erythrocyte sedimentation rate; CRP, C-reactive protein, RRT, renal replacement therapy; SOFA, sequential organ failure assessment. Notes: * Fisher’s exact test; # Chi-square test.

**Table 2:** Factors that predict the development of third stage acute kidney injury.

Characteristics	Univariant Analysis OR (95% CI)	*p* Value	Multivariant Analysis OR (95% CI)	*p* Value
Age in month	0.993 (0.988–0.998)	0.003	0.990 (0.981–0.997)	0.011
Leukocytosis	2.05 (1.02–4.10)	0.043	2.17 (0.89–5.28)	0.089
Thrombocytopenia	3.38 (1.24–9.18)	0.017	1.02 (0.27–3.77)	0.978
+ve blood culture	2.87 (1.79–4.60)	<0.001	2.2 (1.1–4.6)	0.026
+ve CSF culture	5.96 (1.09–1.87)	0.019	Omitted *	-
Diuretics	2.53 (1.55–4.14)	<0.001	0.81 (0.35–1.86)	0.614
Inotropic support	3.11 (1.82–5.32)	<0.001	0.81 (0.65–3.68)	0.351
Amikacin	2.65 (1.54–4.55)	<0.001	1.99 (0.93–4.56)	0.077
Gentamycin	3.24 (1.55–6.77)	0.002	2.89 (1.04–8.05)	0.042
Vancomycin	2.79 (1.72–4.53)	<0.001	1.29 (0.64–2.59)	0.474
SOFA	1.23 (1.12–1.34)	<0.001	1.22 (1.08–1.37)	0.001
Anemia	2.37 (1.31–4.29)	0.004	1.32 (0.37–4.69)	0.665

Abbreviations: OR, odds ratio; CI, confidence intervals, CSF, cerebrospinal fluid, SOFA, sequential organ failure assessment. * +ve CSF culture != 0 predicts success perfectly.

## Data Availability

Upon available request to corresponding authors.
